# Stressors for farmworker parents during wildfire season

**DOI:** 10.1186/s12889-024-20671-x

**Published:** 2024-11-28

**Authors:** Savannah M D’Evelyn, Isabel Nerenberg, Maria Blancas, Laura Rivera, Alyssa Suarez, Dennise O Drury, Edward J Kasner

**Affiliations:** 1 Department of Environmental and Occupational Health Sciences, University of Washington, Seattle, WA USA; 2 Pacific Northwest Agricultural Safety and Health Center, University of Washington, Seattle, WA USA; 3 The Community for the Advancement of Family Education, Wenatchee, WA USA

**Keywords:** Farmworker parents, Wildfire smoke, Air pollution, Messaging, Communication, Northcentral Washington

## Abstract

**Background:**

The severity of wildfire seasons amplify stressors that farmworker families in the Pacific Northwest face as they balance childcare, work, and personal wellbeing. A lack of safe and attainable childcare has been a challenge for farmworker parents since before the Covid-19 pandemic and is of particular concern during wildfire season when parents must weigh the risks and benefits of leaving children at home, taking them to work, or sending them to childcare. This study describes how stressors of balancing childcare, work, and concerns about children’s exposure to smoke during wildfire season impact the wellbeing and workplace absenteeism and presenteeism for farmworker parents.

**Methods:**

To understand the impact of this balancing act on farmworker parents, researchers from the University of Washington partnered with Wenatchee’s Community for the Advancement of Family Education to conduct interviews with 20 farmworker parents, and co-host two town hall discussion meetings within the community.

**Results:**

Six qualitative themes emerged from our interviews including that farmworker parents feel both ill-prepared at home to protect their families, and also do not feel as though they are being provided with adequate resources at work to protect their own personal health. Through the town hall events, we learned that verbal messaging and storytelling are effective and appreciated routes of communication.

**Conclusions:**

Overall, we found that messaging and effective communication around how to prepare for the worsening levels of smoke is lacking among the farmworker community. Future research will address the messaging and communication gaps that must be filled to protect the health of both workers and their families during smoke season and beyond.

**Supplementary Information:**

The online version contains supplementary material available at 10.1186/s12889-024-20671-x.

## Background

Wildfire smoke season in the Pacific Northwest has increased in duration and intensity over the past decade [1]. Wildfires in north central Washington consistently account for more than 50% of the acres burned across the state [2]. In addition to local fires, the geography of this region funnels smoke into valleys such that residents are also regularly inundated with smoke from other parts of the state and country. In 2021, air quality in Okanogan County was moderate - or an Air Quality Index (AQI) of 51 or higher for over half of the summer months. This included two full weeks of air that was either unhealthy for sensitive populations (including children, the elderly and outdoor workers) or worse. The maximum AQI reached 240 [3]. According to the US EPA, an AQI of 51–100, means air quality is acceptable, however, there may be a health risk for people unusually sensitive to air pollution [4, 5]. An AQI of 240 is unhealthy for everyone who is exposed.

Outdoor workers are considered sensitive populations under the AQI definitions because they cannot avoid exposure. In a recent study by Wood et al., rural residents were asked what steps they take to protect themselves and their families during a smoky day. The most common response from interview participants was to remain indoors [6]. Factors including language barriers, access to transportation, immigration status, and access to healthcare are just a few of the social determinants of health that affect seasonal and migrant farmworkers across the Western US [7–9]. Due to the nature of their work, and their inability to escape exposure by heading indoors, and insufficient clean air indoors, farmworkers are exposed to higher-than-average levels of smoke throughout the wildfire season [10]. Smoke also infiltrates poorly insulated farmworker housing through small openings and cracks around windows and doors [11], making it impossible for workers and their families to escape smoke entirely. Agricultural workers in particular are over-exposed compared to other outdoor workers, as their work does not stop during wildfire season, but rather intensifies during the coinciding harvest in summer and fall. This higher workload also leads to a potentially higher dose of smoke inhalation with exertion and increased respiratory rates with increased physical labor [12–14]. Studies across the western United States have documented a disproportionate burden of wildfires both on farmworkers and their families [10, 15–17].

Direct exposure to wildfire smoke can lead to acute symptoms such as wheezing, coughing, headaches or irritation of the nose, eyes, and throat [18–20]. Chronic exposures experienced by outdoor workers in the summertime can include increased exacerbation of respiratory diseases such as asthma or chronic obstructive pulmonary disorder (COPD), along with increased risk of cardiovascular disease and other cardiopulmonary conditions [21–25]. In addition to the impacts of direct exposure, farmworkers may carry particulates home on their clothing after working outdoors during periods of heavy smoke exposure. Past studies have found that clothing can serve as a reservoir for chemical pollutants and particulates [26], requiring further action by farmworkers to prevent indoor exposure, including wiping shoes with a wet towel and removing clothing before entering their homes [27].

Data from the Oregon Covid-19 Farmworker Study found that farmworkers are more concerned about their family’s exposure than their own [28, 29]. This worry is intensified when farmworker parents are not able to access adequate childcare options for their children - particularly during fire season. During the Covid-19 pandemic, many working parents struggled to find adequate childcare for their families [28, 30–33]. For farmworkers in particular, limited childcare led some parents to bring their children into the field or leave them at home with a sibling or other relative [28, 30, 31]. However, for farmworker parents, finding accessible and affordable childcare options has been a challenge since long before the pandemic [17]. In a 2017 study, researchers found that in families where both parents are working outdoors, there was a desire to move to work where childcare would be available [17, 34]. Surveys of 102 agricultural business owners found that a major barrier to communication around childcare was that businesses lacked guidance on how to provide childcare options for their workers [34].

In a survey of farmworkers across California, researchers found that knowledge of the health impacts and preparedness for wildfire smoke, including knowing and having the ability to take action to mitigate health impacts on poor air quality days, varied widely across farmworker groups [10]. This survey addressed perceptions for farmworkers themselves but did not investigate knowledge or perceptions around family exposures. Adaptations are made by farmworker parents who do not have optimal access to childcare that may impact children’s exposure to air pollutants [30, 35]. We hypothesized that during wildfire season concern for children’s exposure to smoke may impact farmworker wellbeing as they balance work, childcare, concern for their own exposure, and concern for their family’s exposure. This study aimed to understand the impact of this balancing act on farmworker parents, and worked to identify resources and tools to help families adapt to the ever-worsening wildfire seasons.

## Methods

### Study aims

While several studies have investigated the impact of wildfires and wildfire smoke on farmworker populations, few studies have explored the impact of wildfire smoke specifically on farmworker families. The aims of this study were to first, describe the impact on farmworker parents of balancing childcare with both work and concerns about their children’s health related specifically to wildfire smoke exposure, and second to propose potential solutions to mitigate the stress of worrying about children’s exposure through safe and attainable childcare.

The aims were completed through key informant interviews with farmworker parents. These interviews provided details on the overall state of farmworker parent wellbeing during fire and smoke season. We chose to follow the interviews up with two local townhall events to give the regional farmworker parent community a chance to reflect on what we learned from interviews and be a part of brainstorming solutions moving forward.

### Community partnership

Wenatchee Community for the Advancement of Family Education (CAFÉ) works with widespread, culturally-diverse communities in north central Washington to provide opportunities in leadership, civic and social engagement, literacy development, and academic advancement. With the increasingly severe wildfires in their area, CAFÉ recently took on wildfire adaptation as a topic of interest and has since piloted and established a five-week course on wildfire preparedness and education for community members across the region. Community members who are inspired by this course have the opportunity to join CAFÉ’s Alianza Ambiental (Environmental Alliance). This group meets on a monthly basis to discuss how to protect their community from wildfire and wildfire smoke. This wildfire specific priority, along with CAFÉ’s goal to reach a broader farmworker community aligned closely with the goals of this study.

The Pacific Northwest Agricultural Safety and Health Center (PNASH) and the University of Washington partnered with a Alianza Ambiental to complete this study as community-based participatory research (CBPR). The chair of Alianza Ambiental, a CAFÉ staff member, was involved in every stage of this project after the initial grant writing. A joint UW and CAFÉ team worked together to write and translate the interview guide (Appendix A), and after the initial transcript coding process, the CAFÉ team was consulted to identify final themes. The CAFÉ team was involved in developing the agenda for the townhalls and served as joint facilitators alongside UW team members.

### Study timeline

This study was conducted over the spring and summer of 2022. Recruitment began in the spring and continued until the week before the townhall events. All key informant interviews were conducted between April and July. Initial analysis of the interview data occurred before the townhall events which both occurred in mid-August. Final analysis of the combined data collected from both interviews and townhalls occurred in the fall and winter of 2022. Throughout this project, no serious wildfires or smoke events impacted either the Wenatchee or Okanogan areas, and thus current fire and smoke were not taken into consideration as far as having an impact on data collection.

### Recruitment

#### Key informant interviews

Alianza Ambiental steering committee members recruited interview participants during January and February of 2022 utilizing the committee’s existing connections with the Chelan and Okanogan County agricultural communities. Inclusion criteria for interview participants included (1) participants must currently or previously have worked in agriculture within Chelan or Okanogan Counties, and (2) participants must currently have children under the age of 12 years, requiring them to procure some form of childcare during the summer months. Flyers describing the project’s methods and intentions were distributed via the CAFÉ newsletter and social media posts via Facebook and Instagram. Additional outreach occurred via telephone and through the La Pera Radio program.

Recruitment for participation in the town halls was conducted by the UW research team, PNASH Outreach team, and Alianza Ambiental steering committee members. Recruitment began during the key informant interviews and continued up until the dates of the town halls in August of 2022. Participants and resource organizations (Appendix B) were primarily contacted via phone, Whatsapp, and social media. For both townhalls combined,183 participants were invited, and approximately 30–40 farmworker parents attended each event.

### Data collection

UW IRB approval was acquired before participants were contacted, and all participants provided verbal and/or written consent prior to their participation. All key informant interview participants were asked to fill out a demographics survey (Appendix C) to ensure that inclusion criteria was met. A total of 20 interviews were conducted with farmworker parents; ten located in Chelan County and 10 located in Okanogan County. Each interview participant received a $50 gift card as compensation for their participation, as well as additional health and safety information on wildfire smoke. Each interview lasted between 20 and 45 min, and was audio recorded through Zoom or Google Voice. Interviews were transcribed using TranscribeMe and translated by Alba Enterprises Language Connection. While there was no participant member checking of the interview transcripts, both the UW and CAFÉ team checked the transcripts for contextual accuracy.

Town Halls were hosted at the Chelan County Pybus Public Market and the Okanogan County Fairgrounds in August 2022. Both events were set up so that separate time and space was available at the beginning and end of each town hall for participants to visit and learn from the tabled resource organizations. After tabling and introductions, the events began with an overview of the interview findings, followed by a facilitated panel with previously identified interview participants who were willing to share specific stories that had come up during their interviews.

The second half of the meeting consisted of round table discussions between four to seven farmworker parent participants, an Alianza Ambiental or Spanish-speaking PNASH team member, and a note-taker. Town hall participants self-selected into roundtable groups based on one of three themes. Participants were encouraged to sit with people they did not already know. The goal of this portion of the townhall events was to get initial feedback on the analysis of interview results from additional farmworker parents and encourage further discussion. Due to time constraints and the larger number of participants, it was not possible to get specific feedback on every single theme. Thus, the original expected topics of healthcare, childcare and wellbeing were slightly broadened (based on the themes that emerged) and turned into the three discussion topics for the roundtable discussions: (1) Health Effects of Smoke Exposure and How to Protect One’s Health (Themes 1 & 2); (2) Access to, Reliability of and Comfort with Childcare (Theme 3); and (3) Stress and Mental Wellbeing (Themes 4 & 5). Roundtable discussion questions were co-developed in advance by the UW study team and key members of Alianza Ambiental (Appendix D). All questions were facilitated using a combination of the Liberating Structures facilitation techniques *Conversation Cafe* and *15% Solutions* [36]. The *Conversation Café* was selected to promote participation from all group members, and to give time for both reflection on each other’s and their own experiences. *15% Solutions* is a technique that encourages participants to think about what they could accomplish right now with the resources available to them and brainstorm possible solutions to current challenges.

Several team members from both UW and Alianza Ambiental were designated as notetakers to capture date from the day. Each roundtable group consisted of one facilitator and one Spanish-speaking notetaker that was transcribing participant responses to the discussion questions. All note takers were given printed copies of the discussion questions with space below and instructed to take bulled hand-written notes. After the townhall a UW team member transcribed all notes and followed up with notetakers and facilitators for clarification as needed.

### Analysis

Data analysis was conducted by a combined team of PNASH researchers, UW public health students and members of the Alianza Ambiental team who did not conduct interviews. All transcripts were blinded. While members of the research team have worked in this region of Washington, this particular community of farmworker parents was a new study population to everyone on the UW team. Certain members of the Alianza Ambiental team had close relationships with the farmworker parents interviewed or had previously worked as farmworkers themselves. We believe that this perspective added richness to the interviews themselves, but asked these team members to recuse themselves from the analysis portion of the project.

A codebook for analysis of interview transcripts was developed using both deductive and inductive approaches. Expected topics of healthcare, childcare, and wellbeing (developed from the research question and then the interview guide) were used to construct primary and secondary codes. Primary categories included children’s exposure to smoke, worker protections, wildfire smoke perceptions, stress, childcare options. Three randomly-chosen transcripts were dual-coded by two members of the UW project team to both increase the level of inter-coder reliability and allow for initial revisions to the codebook definitions. The remaining transcripts were coded by one of the two UW coders. Both coders used NVivo qualitative analysis software.

The Framework Method [37] of thematic analysis was used throughout the data collection and analysis process. First, codes were developed based on a set of predetermined topics around the research question and thus within the interview guide. After data collection through key informant interviews, transcripts were coded to primary and secondary codes to identify key themes. Coded transcript quotes were charted into a matrix to determine frequency and distribution of data and then mapped to allow for easier interpretation of the data. To assure the transcriptional and cultural accuracy of these thematic findings, opportunities for feedback from CAFÉ team members were integrated into each step of the analysis process. As new themes were identified by UW team members, each was presented and discussed with the Alianza Ambiental chair to validate the accuracy of the findings.

Notes taken during the roundtable discussions were not as consistent as the interview transcripts, as notetakers were determined the day of and not given explicit instructions other than to write bulleted responses to each discussion question. Additionally, tangents and storytelling were encouraged. All notes were roughly charted into the original themes derived from the key informant interviews using a charting and matrixing technique as described above. Two additional themes were identified based on town hall notes and quotes that did not fit into the original matrix.

## Results

### Themes

Over the course of the interview process, we asked participants to describe their perceptions of wildfire smoke impact on the health and well-being of their families as well as the impact that inconsistent childcare has on farm worker parents’ well-being. The themes and subthemes that emerged from these interviews are presented in Table 1.

**Table 1:** Themes from key informant interviews

Theme	Subtheme
1. There is a need for more resources to prepare farmworkers and their homes for smoke events	1.1 Farmworkers do not feel prepared to protect themselves outside of their homes
2. There is a need for more resources to prepare worksites for smoke events	2.1 Consistency of resources provided to farmworkers by employers is sporadic
	2.2 There are many instances of smoke exposure on worksites
3. There is a need for more accessible summer childcare programs	3.1 Farmworker parents are concerned about their children’s health and the preparedness of their childcare during emergencies
4. Worksite responses to smoke are unpredictable	4.1 Decisions made during smoke events often lead farmworker parents to be concerned about financial instability
5. There is a need to find ways to help families cope with the stress of smoke events	5.1 Farmworker parents are concerned about the mental wellbeing of their children during smoke events
	5.2 Farmworker parents struggle with their own mental wellbeing during smoke events
6. There is a need for smoke safety related information for farmworker parents	

Subsequent analysis of town hall discussion notes was also conducted via an abridged framework method. This analysis led to the addition of two themes listed in Table 2.

**Table 2:** Themes from town hall roundtable discussions

7. There is a need for improved real time messaging	7.1 Current evacuation notices are not reaching the farmworker community
	7.2 Real-time air quality warnings are needed
8. Farmworker parents need a space to share stories around wildfire and other climate-related experiences	

As previously mentioned, the initial expected topics and analysis of the themes that emerged from farmworker parent interviews led us to design the townhall around the three subject matter topics of (1) Health Effects of Smoke Exposure and How to Protect One’s Health (Themes 1 & 2); (2) Access to, Reliability of, and Comfort with Childcare (Theme 3); and (3) Stress and Mental Wellbeing (Themes 4 & 5). The need for improved communication behind Themes 6 and 7 was integrated into all three discussion categories. To be inclusive of both the interviews and the townhalls, the following descriptive results are organized into these three categories.

### Health effects of smoke exposure and how to protect one’s health

#### Themes 1 and 2

When asked what they knew about the health impacts of smoke exposure, the majority of farmworker parents talked from experience rather than information they had learned or been taught. Themes 1 & 2 speak to the need for additional information and resources to prepare for smoke exposure and mitigate the impacts of exposure on farmworkers and their family’s health.

I don’t really know much about it. I think the smoke causes if you breathe black smoke, it causes breathe problems to your lungs, and you start coughing a lot. - Chelan Farmworker Interviewee.

I’ve always known that smoke does harm your health, that you must be breathing in all that smoke, it does bad.” - Chelan Farmworker Interviewee.

“Yes, because it is pure harm to us.” - Chelan Farmworker Interviewee.

In one manner or another, every participant indicated that they did not feel prepared to protect themselves and their families from smoke. Many participants stated that although they do what they can to smoke-proof their homes, in many cases, the quality of material and infrastructure (e.g., crevices or openings under windows and doors) allow smoke to infiltrate during particularly heavy or long-lasting smoke events. Additionally, participants stated there are financial and resource based barriers to making improvements that are often prohibitive.

I like to have them out here as much as I can; but I’m also aware that the windows in my house aren’t so safe and we can’t do everything. In other words, I would prefer to change the windows, I would secure everything so that they would be well protected in here; but as I say, one is not enough to do everything. - Chelan Farmworker Interviewee.

It is difficult too, because when there have been fires and there is smoke here, then we do not go out and still smoke gets in through the windows. And you go out with masks and all that and even with that you breathe it anyways. - Chelan Farmworker Interviewee.

I keep thinking about the impact that fires have on the communities. And how we are – we’re not, sorry, prepared enough for these fires. - Okanogan Farmworker Interviewee.

I think we need more how to protect ourselves from the smoke and what we can use, like a safer mask or procedures. - Chelan Farmworker Interviewee.

Several participants stated that having access to appropriate masks or portable air cleaners would help them to feel more comfortable and safer during smoke events. However, access to N-95 masks are limited - especially in rural areas [38] and many portable air cleaners are not financially feasible for farmworker families. Even though employers were required to provide respiratory protection per the Washington Labor and Industries (L&I) Wildfire Smoke (Emergency Rule) at an AQI of 101 from June through September of 2022 [39], participants reported that resources provided by employers during smoke events varied greatly. Variations included everyone being provided with surgical masks – which do not protect against PM2.5 exposure – only specific workers given masks, no masks were provided at all, or farmworkers were asked to bring their own. Several participants stated that surgical masks provided were originally for Covid-19, and new masks were not provided during smoke events. Other participants stated that masks were only provided if farmworkers specifically asked for them. Farmworkers reported that none of the employers of interviewed farmworkers provided N-95 respirators during smoke events.

I believe that there should be specifications, or they should force employers to provide. Last year they provided masks because of COVID, but for people, they didn’t give them to everyone. Almost everyone had to bring their own mask because of COVID. But in terms of smoke, I have not seen them say: “I am going to provide you with the mask so that you can use it during this time. -Chelan Farmworker Interviewee.

He gave my husband one of the masks, for example, the N95 when the COVID thing happened, but he was the manager. But to the others, well, everyone needs to bring their own [laughs]. - Chelan Farmworker Interviewee.

Information shared on the worksite about the health impacts of smoke exposure was limited, and participants stated that information was only shared with farmworkers in written form. When asked about what resources would be helpful on the worksite, participants suggested that training for all employees about smoke safety and the consequences of smoke exposure could lead to implementation of safety rules for farmworkers.

A lot of times they’re not – not the crew bosses, because they follow their supervisor’s instructions, but I think companies have to start there, educate them, the damage they’re doing to the people when they bring them to work. - Okanogan Farmworker Interviewee.

For them to provide us with what we need for the smoke and more information so we know. - Okanogan Farmworker Interviewee.

No, that they give us protection, that they give us the masks. And that they give us information about smoke and that we had a way to measure the smoke, because it has a certain degree that, you know? – what is it called? When you don’t have enough air? When the smoke is very strong. - Okanogan Farmworker Interviewee.

### Access to, reliability of and comfort with childcare

#### Theme 3

Theme 3 outlined a need for more accessible summer childcare programs which was echoed by the majority of farmworker parents during the townhall events. Participants stated there was a lack of suitable childcare options in Chelan and Okanogan counties that care for children of different ages, are easily accessible for parents both geographically and financially, and were prepared to keep children safe indoors during a smoke event. Parents described fear that they could not trust their current options in case of a fire or smoke emergency. Concerns over the preparedness were primarily focused on the concern that childcare providers were allowing children to go on with their normal activities during smoke events (e.g., going out to recess and playing outside, etc.) or their lack of capacity to care for the children in emergency situations.

According to all participants - both interviewees and townhall participants - there were no employer sponsored childcare programs available. During the townhall in Okanogan county, some farmworker parents also expressed how changes in their pay impacted their benefits, such as the ability for them to qualify for childcare assistance programs. The changes in their pay were small and only for the season, yet they would lose their benefits and no longer be able to afford childcare. This often occurred during the summer - the busiest time for harvest – which often overlapped with smoke events.

I think so, I think that a lot of the parents here in the area would love to have a more reliable option, an option they can count on and say, “This is my childcare, it’s not going to fail me, unless my kids get sick and I have to stay at home with them.” If we need it, it would be important for many of these families to have that support. - Okanogan Farmworker Interviewee.

I think it really depends. Obviously, when you have government daycare facilities that are run by people in small towns, they obviously have protocols that they need to go through and stuff like that and don’t allow the children to be outside. I feel like it’s a little bit more difficult for people who have low income or just can’t afford the fees that it is to take your children to daycare. And so you’re forced to kind of find someone else that’s secondary to take care of your children. It becomes a little bit harder because it’s not someone– they don’t have those set protocols that they have to follow. - Chelan Farmworker Interviewee.

And our children the same, because at school the children go out to recess and inhale all that smoke that is so dangerous. - Chelan Farmworker Interviewee.

If there was a way we could be sure that the person or daycare, who is taking care of our children, is trained to act in case they are exposed to smoke, if they know the protocol, if they have their daycare or their house equipped in case of smoke, do you understand me? If they have filters, if they have any– they blocked the windows. They put rags on them -- to those who can’t have filters, they put some rags on them. That is, to know if really in the places where they are taking care of our children, that there is a way to know. - Okanogan Farmworker Interviewee.

Yes, because you cannot watch out for their health. You do not know what is happening with your children where they are, where you have left them, whether it is daycare or in your own home. In the care of a brother, who may not even know what to do. - Chelan Farmworker Interviewee.

Farmworker parents observed increased stress in their children during smoke events when they were told they needed to remain indoors for their health and safety. Parents stated this stress came from the fact that their children were no longer able to participate in the activities they normally would during the summer, and additionally that being sequestered in an enclosed environment during an emergency could be traumatic and cause fear. Participants described the deterioration of their children’s mental well-being during these times from being bored, stressed, listless, to fearful.

And that does affect them, being outside is not the same as being inside. But psychologically sometimes they do depress me. - Chelan Farmworker Interviewee.

Yes, because they are used to playing outside in the summer, most of the time, outside, being free. And when it’s burning, it’s obvious that I don’t let them out and sometimes they feel bored and stressed. - Chelan Farmworker Interviewee.

Yes. They worried; they always worry. They say: “Mommy, isn’t the fire going to get here? We aren’t going to get burned, right?” They do worry, they stress, Aren’t we going to get burned? “they say. “No. they’re going to put out the fire. - Chelan Farmworker Interviewee.

Due to the timing of this study, some of the stated difficulties surrounding access to childcare stemmed from lingering Covid-19 closures. However, multiple studies in addition to our own identified that finding adequate childcare was a challenge for farmworkers even before the pandemic [17, 34]. Our interview questions specifically asked parents about their childcare situation before and after the pandemic, how things had changed and what their continued concerns might be. Much of the conversation around childcare during the townhalls did not center on access itself, but parent’s comfort level with their current situation, and if they believed their children would be safe during a smoke or fire event. In addition to the workplace, locations with vulnerable populations such as schools and childcare centers need more information around how to protect these populations during a wildfire event.

### Stress and wellbeing

#### Themes 4 and 5

Theme 5 spoke to a need for more ways for both parents and their children to deal with the stress of fire season. Farmworkers stated that the number one cause of stress during smoke events was the stress of having to leave their children at home or at daycare with someone else while they were at work. For some participants, this impacted their focus at work, with some describing leaving their workspace multiple times during a shift to call and check up on family. While concern over their children’s safety was not unique to smoke events, the emergency event status exacerbates the impacts of stress.

And that’s what I say, it’s so difficult, really, to work and at the same time have children, because you don’t feel at ease.” - Chelan Farmworker Interviewee.

If suddenly something happened, and it was something that neither she nor we knew what to do while my children were with her and I working. - Okanogan Farmworker Interviewee.

I don’t feel prepared or qualified to tell you. When it is more than 15 days of smoke, not only do the children get stressed, or your children are stressed, but you already begin to stress yourself too.” - Chelan Farmworker Interviewee.

But it is worrying that at school maybe they will take them out to recess, you know the children, as if nothing happened. - Chelan Farmworker Interviewee.

Last year there were very serious fires, I had to be working at night on the packaging, my husband was at home with the children, and I asked permission to go to the bathroom and at that time I would talk to my husband and say: “You know what? Here inside the packing house, it is very smoky, the air is very saturated of smoke, we are very close to the fire. How are you there? How are they? - Chelan Farmworker Interviewee.

Farmworker well-being was also impacted by the unpredictability of worksite response to smoke and fire events (Theme 4). Participants described a variety of situations that occurred at worksites during smoke events, including instances of worksite closure until the smoke level was less severe, employee choice to take time off or continue working through the smoke event, as well as required continuation of work through the smoke event despite dangerous air quality levels. The lack of consistency in worksite responses forced farmworkers to make the choice to either compromise their health and their family’s safety during smoke events or stay at work to maintain financial stability.

Yes, because sometimes we are working and the smoke is very heavy. And yes, sometimes they provide us with masks, but sometimes they don’t. - Okanogan Farmworker Interviewee.

Sometimes when there is too much smoke, when you can’t even see, we stop working a day or two, it depends on how it is. - Chelan Farmworker Interviewee.

I mean, it’s funny, because when it’s very, very, very hot, they do stop the picking or they stop the activity, because they say: " It’s too much”. if it is very hot. Or when it is very cold, they cannot go to work if the temperature is very low. And yes, they do that. But, when it comes to fires, which goes directly to the lung and is damaging -well, it can affect your performance if you don’t breathe well-, I haven’t seen them stop the activity. - Chelan Farmworker Interviewee.

During townhall discussions, several farmworkers commented on the fact that if they chose to take time off during smoke events, there was the fear that they would be fired or replaced. When the worksites remained open and operative during smoke events, participants described their concern that the long exposure to smoke may impact their health or their ability to care for their families during smoke events. Several participants also described instances in which worksites were kept open during smoke events, the employers adopted a “put on a mask and carry on” and “finish the job” mentality. When farmworkers were asked if they felt comfortable asking their employers for additional resources to protect their health from smoke exposure, everyone responded “no”. When facilitators asked during the townhall if there was a fear of retaliation if they were to ask for additional protective resources, all farmworkers raised their hands in agreement.

And another thing, when I’m working on the warehouse, the doors are open, because the forklifts go in and out. Most of the day are open. And the fires there nearby, because it is pure pine around there in the work area. And the fires are nearby, because around the work area there is just pines. Then, the smoke goes all the way inside, you can see it; as the sunbeams enter, because the packing has the fans and all that, yes, it is possible to detect all the smoke and all that. Imagine, not even inside the packing we are safe, now imagine out here when we leave. - Chelan Farmworker Interviewee.

I think, as you said, the childcare. I think that if the smoke extends for a long time in which we are going to be exposed, it would be that if you could make the parent stay at home and care for his children, for the sake of his children, and to know that it is a valid reason at work, without having to be worried about: “They are going to fire me because I have not been going to work for five days, because I would rather be taking care of my children and be safe and sound, and protected inside the house, instead of waking them up early in the morning to take them to a daycare, exposing them to smoke. And vice versa, bringing them home and expose them to smoke.” - Okanogan Farmworker Interviewee.

Unfortunately, in the field there are many people who just tell them, “Put on a mask and carry on. - Okanogan Farmworker Interviewee.

Because sometimes they are just interested in us finishing the job. And they know that you are a father, but they still want us to go to work and get the work done. - Chelan Farmworker Interviewee.

The lack of consistency in worksite response could also impact the financial stability of farmworkers (Theme 4). If a participant felt unsafe at the worksite during a smoke event and felt comfortable to request to leave, most worksites allowed them to take that requested time off, but unpaid. Some participants described that they continued to work through smoke events because of this financial instability despite their understanding of the potential health effects of smoke exposure, referencing their need to pay bills and provide enough for their families. As smoke events are lasting longer, this financial dilemma is becoming more of a burden to farmworker parents.

So, I have known that the employer or some of the employer helps co-workers to let them go to take care of their child, respecting them, maybe not paying them, but respecting their job, that is, keeping their work and not losing it. - Chelan Farmworker Interviewee.

But at work, you know that there are risks that you must take, but at the same time it is necessary because we must work, because if we do not work, we do not have money to pay the bills or the rent. - Chelan Farmworker Interviewee.

## Discussion

In this study, farmworkers reported that concern for children’s exposure to smoke impacts their wellbeing as they balance work, childcare, concern for their own exposure, and concern for their family’s exposure. Farmworker parents feel that they do not have the adequate tools and resources to keep themselves and their children safe during smoke events. Through this study we were able to identify gaps in communication, resources, and messaging that need to be addressed in order to overcome this challenge.

### Informational needs

Outdoor workers are exposed to wildfire smoke more than most other populations in this community [40], and they must have the information necessary to keep themselves safe during smoke events. During the townhall event, several farmworker parents mentioned that this setting was the first time anyone had talked to them about the air quality index and the recommendations provided for each level to protect one’s health. The emergency ruling by Washington L&I that was in place from June through September of 2022 stated that employers must provide their employees with effective information and training on the implications of exposure to levels of PM_2.5_ above 69 AQI [39]. Farmworkers stated that if any information was shared at all, it was in a pamphlet posted on a bulletin board, and more likely than not, in English. Since the end of this study, Washington L&I has put in a permanent Wildfire Smoke Rule for Agriculture [41].

The current lack of accessibility to needed information is putting farmworkers and their families at risk. This was made clear during the townhall events when multiple discussion groups side-tracked their conversations to discuss the stress of not only smoke, but fire danger. In this region of Washington, individuals must sign up for emergency notifications or they will not be received. Additionally, when they do receive them, they are in English. To protect communities in emergency situations, messaging should be easily accessible and not have to be translated by the recipient to know if they need to evacuate their children to a safe location.

Throughout the interviews and focus groups, participants shared that they did not feel that they could request additional tools or resources from their employers for fear of retaliation. This shows a need to improve the social capital and autonomy that farmworkers have at their worksite. Improved worksite environments so that the response to a smoke event is clear and consistent would reduce at least one category of stress for farmworker parents [39]. A new permanent rule for Wildfire Smoke in agriculture went into place in January 2024 after the close of this study, however, similar to the emergency ruling that was in place at the time of the study these protections would need to be effectively implemented and enforced in order to be truly protective. More coordination at the state and county levels is needed in response to these events. Farmworker responses demonstrated that workplaces had very different responses in each county and that employers did not have a clear direction as far as what their role versus the role of the county was.

The need for improved information and communication was repeated throughout the interviews and townhall events. Farmworker parents not only did not know what to expect in protections from their workplace, but they were also uninformed when it came to what the health impacts of repeated smoke exposure could be for them and their families. This information should and could be common knowledge across this community if appropriately shared.

### The role of CBOs

During the COVID-19 pandemic, frontline community-based organizations (CBOs) across Washington organized to protect and advocate for farmworkers and other at-risk populations. CBOs recognized the importance of networking, coalescing, and addressing the ongoing needs in their communities. These groups are working to improve messaging and communication around fire and smoke and are the on the ground players when it comes to providing resources. During the townhall events, various CBOs were invited to share information, contact information and resources with farmworkers. CAFÉ passed out box fan filter kits and information about how to use them in your home, and other organizations distributed N95 masks. These events were incredibly well received for both the access to resources, as well as the ability to learn and discuss community needs in person. A group of farmworkers from Okanogan County working on a wildfire safety course with CAFÉ decided to organize a similar event among their peers to share what they had learned.

The power of CBOs to provide needed information and resources to their community is unmatched. The relationship that CAFÉ has established with their community allowed for the successful recruitment of 20 interviewees and two townhall events that were attended to capacity. CAFÉ hosting and facilitating the townhall event was powerful as it fostered a safe space for participants to share meaningfully. CAFÉ’s location, trust, and advocacy was key for creating meaningful change. This level of engagement would not have been possible with only members of the University of Washington team. Researchers seeking to establish more meaningful relationships and creating an impact that extends beyond the life of the project should consider working with a CBO that has the ability and capacity to take a leading role in the community engagement aspect of the project.

### Strengths and limitations

Recruitment of farmworker parents for interviews was done entirely by staff at Wenatchee CAFÉ within the Alianza Ambiental Steering committee, and therefore the pool was limited to those that had primary or secondary connections to the organization. By the time 20 interviews were completed, data saturation had been reached, however it is always necessary to acknowledge the potential that not all perspectives may have been represented. This limitation was somewhat overcome during the townhall events where recruitment was opened up beyond Wenatchee CAFÉ. Flyers and cards were distributed in person and on social media in order to recruit community voices that may not have been included in the interviews.

The limitation of having Alianza Ambiental recruit all participants for interviews became a strength in the interview process itself. Farmworkers were able to be interviewed in their native language by Alianza Ambiental members that they had already established a trusted relationship with. The townhall round table discussions were facilitated by Alianza Ambiental members, providing a level of comfort for participants that would not have been achieved if being conducted by the UW team. Despite the overall positive aspect of community members serving as the participant-facing team members, there is some potential for social desirability bias to consider, and that participants may be selectively answering interview questions with what is expected or to better fit the community norm. To counteract this, the open communication of the townhalls provided a more comfortable space to discuss difficult topics, making it less likely that participants would feel uncomfortable voicing their thoughts. In addition to this, all Alianza Ambiental steering committee members and participant-facing team members were Spanish speakers, allowing easier communication for participants throughout their interviews and townhall discussions.

## Conclusions

When asked if they had worked during a day that was so smoky they could not see the sun, every parent in the room raised their hand. Farmworker families in north-central Washington are severely impacted by wildfires, smoke exposure, and extreme heat. Despite this, engagement with these communities on how to mitigate the health effects of these climate impacts is currently low. Our findings indicate that not only do farmworker parents not feel well educated or informed on the health impacts of smoke exposure, they are also concerned that both their homes and workplaces lack sufficient resources to keep themselves or their families safe when smoke is on the rise. Effective messaging around wildfires and smoke either does not exist, is inaccessible, is not translated into the primary language of workers, is not shared through appropriate channels, or it is not culturally responsive. Future work is needed to improve both the access to and content of wildfire communications and resources to keep farmworkers and their families safe during wildfire season and beyond.

## Electronic supplementary material

Below is the link to the electronic supplementary material.

Supplementary Material 1

Supplementary Material 2

Supplementary Material 3

Supplementary Material 4

Supplementary Material 5

## Data Availability

The data that support the findings of this study are housed at the University of Washington. Restrictions apply to the availability of these data, which were used under license for the current study, and so are not publicly available. Data are, however, available from the corresponding author upon reasonable request and with permission of the University of Washington.

## Abbreviations

AQI: Air Quality Index
﻿CAFÉ: The Community for the Advancement of Family Education
CBO: Community-based Organization
PNASH: Pacific Northwest Agricultural Safety and Health Center
