# Supplementary material for: Stressors for farmworker parents during wildfire season
Source: BMC Public Health. 2024 Nov 28;24:3314. doi: 10.1186/s12889-024-20671-x (PMC11603887; doi:10.1186/s12889-024-20671-x)
Supplement: Supplementary file 1 — Supplementary Material 1 [file 12889_2024_20671_MOESM1_ESM.pdf]

Demographics Questionnaire

1. What age range do you fall in?
    a. 18-29
    b. 30-39
    c. 40-49
    d. 50-59
    e. 60+
2. What is your gender identity?
    a. Female
    b. Male
    c. Non-binary/non-conforming
    d. Transgender
    e. Two-spirit
    f. Other
3. What is your racial identity?
    a. American Indian or Alaskan Native
    b. Asian
    c. Black or African American
    d. Native Hawaiian or Other Pacific Islander
    e. Latinx
    f. White
    g. Prefer not to answer.
4. How long have you lived in the Wenatchee and/or Okanogan regions?
    a. Less than 1 year
    b. 1-3 years
    c. 3-5 years
    d. 5+ years
5. How many children do you have? Please list the ages of your children.
    ____
6. Does your employer provide paid childcare leave?
    a. Yes
    b. No
    c. I'm not sure
7. What is your occupation?
    ____
8. Between the months of June and November do you work outdoors or indoors?
    a. I work outdoors only
    b. I work indoors only
    c. I work both indoors and outdoors
    d. other (driving a tractor, truck, deliveries, etc)
