# Supplementary material for: Stressors for farmworker parents during wildfire season
Source: BMC Public Health. 2024 Nov 28;24:3314. doi: 10.1186/s12889-024-20671-x (PMC11603887; doi:10.1186/s12889-024-20671-x)
Supplement: Supplementary file 2 — Supplementary Material 2 [file 12889_2024_20671_MOESM2_ESM.pdf]

Interview Questions:

PART ONE: Identify family dynamics, job responsibilities and understand the current childcare situation for each family.

1. Throughout this interview I'm going to be asking you questions about the impact of wildfire smoke on your job as a farmworker and your role as a parent. To begin, can you tell me a little bit about your family?
   Secondary Questions:
   a. How old are your children?
   b. How long have you worked and lived in the area?
   Goal: identify family dynamics and ages of children -- childcare or school level
   - [ ] Identify family members – how many adults and children live in the home?
   - [ ] Identify if participant lives and/or works in the area and for how long

2. What do you do for a living?
   Secondary Questions:
   a. What crops, products or animal farms do you work on in the summer and fall?
   b. What tasks do you do between June and November?
   Goal: determine job duties
   - [ ] Identify what type of agricultural job participant has
   - [ ] Identify what job tasks participant engages in during wildfire season
   - [ ] Identify if participants job changes throughout the year

3. On a scale of 1-5 do you feel like your role as a parent is supported by your employers and why?
   Secondary Questions:
   a. Does this impact the way you approach finding childcare?
   Goal: prompt discussion on impact of parental duties on worker wellbeing
   - [ ] Identify if employee provides childcare or time off to care for children
   - [ ] Identify if finding childcare increases participant's stress level

4. During the summer of 2021, who took care of your children while you were working?
   Secondary Questions:
   a. Was this childcare option your first choice? Why or why not?
   b. Was this different than before the pandemic, in summer of 2019 or earlier?
   Goal: prompt consideration of children's physical safety during smoke events & understand the current available resources for childcare
   - [ ] Identify who cares for children during the summer months
   - [ ] Identify if childcare options have changed during the pandemic
   - [ ] Identify if parents are pleased with their childcare options or they wish they had a different option

PART TWO: Identify knowledge on the health impacts of wildfire smoke, present concerns about children's health and describe how this impacts parents in the workplace.

5. What do you know about the health impacts of wildfire smoke exposure?
   Secondary Questions:
   a. Where did you learn this?

   Goal: Identify background knowledge on health effects of smoke exposure
   - [ ] Understand the level of knowledge about the health impacts of wildfire smoke
   - [ ] Identify where participant gets their information about the health impacts of wildfire smoke

6. Does wildfire smoke impact your day to day tasks at work?
   Goal: identify concern for personal health and safety related to smoke
   - [ ] Identify if participant is impacted by wildfire smoke at work
   - [ ] Identify if participant is concerned for their own health during smoke events

7. What are employers providing or doing to protect workers from wildfire smoke events?
   Secondary Questions:
   a. Do you feel like you can reach out to your employer for extra protection from smoke, or to request time off during a large smoke event?

   Goal: prompt discussion on workplace policies and culture
   - [ ] Identify if participant feels supported by their employer during wildfire season
   - [ ] Identify measures employer does or does not take to protect outdoor workers from smoke
   - [ ] Understand if participant feels as though more protective measures are needed

8. On a smoky day, how does your family respond?
   Secondary Questions:
   a. What actions do you or they take to protect themselves from the impacts of smoke?

   Goal: identify resources and barriers to action to protect children from exposure
   - [ ] Identify if participant takes protective action to protect themselves or their children from wildfire smoke
   - [ ] If so, identify what these protective measures are

9. How and when does wildfire smoke impact your children?
   Secondary Questions:
   a. Are you concerned for your children's wellbeing during wildfire smoke events? Why or why not?

   Goal: identify concern about children's exposure
   - [ ] Identify if participant believes smoke impacts their children's health
   - [ ] If so, identify how they think smoke impacts their children's health
   - [ ] Identify if participant is concerned for their children's health due to wildfire smoke

10. What resources do you need to feel as though you and your children are safe during smoke events?
    Secondary Questions:
    a. Do you feel like their current childcare situation is adequate to protect them from smoke during large wildfire events?

Goal: Identify ways in which the project could take measurable steps toward improving health and safety

- [ ] Identify if participant feels like their child is safe during wildfire season
- [ ] Identify if this concern is related to their current childcare situation
- [ ] Does the participant have ideas - specific to their childcare situation - that would make them feel like their children were safer during a large smoke event?
