# Supplementary material for: Stressors for farmworker parents during wildfire season
Source: BMC Public Health. 2024 Nov 28;24:3314. doi: 10.1186/s12889-024-20671-x (PMC11603887; doi:10.1186/s12889-024-20671-x)
Supplement: Supplementary file 3 — Supplementary Material 3 [file 12889_2024_20671_MOESM3_ESM.pdf]

<u>Townhall Discussion Questions</u>

1. **Efectos sobre salud**
   → Compartan una experiencia cuando sentían que su salud era impactada por el humo de los incendios.
   → Por favor, tomen un momento para reflexionar sobre lo que oíste - compartir como sienten

   → Que es algo que podrían hacer ahora mismo que pueda disminuir los efectos que el humo de los incendios puede tener en su salud (como el resuello, dolores de cabeza, dolor de garganta)?
      <u>ejemplos:</u> usar mascarilla, cerrar las ventanas y puertos durante un evento del humo, cambiar el aire en su casa con un ventilador.
      <u>pregunta extra:</u> ¿Cual es algo que han hecho o han oído que otra persona ha hecho en esas situaciones?
   → Por favor, reflexionar sobre lo que oíste - compartir como sienten

   → ¿Cuales son las cosas que le gustaría hacer, y que está parándose hacer esas cosas?
      <u>ejemplos:</u> Reemplace los filtros en las unidades de aire acondicionado y ventilación, tenga mejores máscaras y otros equipos de protección cuando salgan, quedarse en casa cuando el humo es demasiado pesado
   → ¿Tienes una reacción o pensamientos sobre lo que otros han compartido?

   → ¿Cuáles son algunas cosas que puedan ayudar para apoyar a su familia durante un evento del humo? (note-taker make a list)

2. **Bienestar mental**
   → Compartir una experiencia cuando sentía que su bienestar mental o el bienestar mental de su familia era impactado por el humo de los incendios.
      <u>ejemplos</u>: Sentirse aburrido o inquieto por tener que quedarse dentro, preocuparse por la cantidad de humo que hay en su lugar de trabajo, preocuparse por sus hijos cuando está en el trabajo
   → Por favor, reflexionar sobre lo que oíste - compartir como sienten

   → Cual es algo que pueden hacer ahora mismo que pueda mejorar el bienestar de usted o de su familia durante un evento de humo?
      <u>ejemplos:</u> Póngase en contacto con su familia para asegúrese de que estén bien, distraerse cuando tenga que quedarse adentro con películas / juegos / etc
   → Por favor, reflexionar sobre lo que oíste - compartir como sienten

   → ¿Cuales son las cosas que le gustaría hacer, y que está parándose hacer esas cosas?
      <u>ejemplos:</u> Tómese un tiempo libre durante los eventos de humo, acceso a servicios de salud mental después de eventos traumáticos, tenga un ambiente de trabajo de apoyo
   → ¿Tienes una reacción o pensamientos sobre lo que otros han compartido?

→ ¿Cuáles son algunas cosas que puedan ayudar para apoyar a su familia durante un evento del humo? (note-taker make a list)

3. **Cuidado de los niños**

→ ¿Cuál es su experiencia con el cuidado de los niños durante el verano?

ejemplos: Guardería familiar o pagada, niñeras, guardería principal en el lugar de trabajo, escuela de verano

pregunta extra: Están cómodos con su cuidado de los niños actualmente? Ese nivel de confort cambia durante un evento de humo? ¿Por qué o por qué no?

→ Por favor, reflexionar sobre lo que oíste - compartir como sienten

→ En un escenario ideal, ¿cómo sería su situación de cuidado de los niños durante un evento de humo de incendios forestales?

ejemplos: ayuda de miembros de la familia en su hogar, guardería profesional, guardería en el lugar de trabajo

pregunta extra: ¿Quién cuidaría a su hijo y en qué tipo de ambiente estaría?

→ Por favor, reflexionar sobre lo que oíste - compartir como sienten

→ ¿Cuáles son las barreras actuales que están dificultando este objetivo?

ejemplos: no tengo dinero para pagar la guardería, la guardería está demasiado lejos de casa o trabajo, mis niños son demasiado jóvenes para quedarse en la guardería.

regunta extra: Si no está contenta con su situación actual de cuidado de los niños, ¿cuáles son sus razones para quedarse con ellos?

→ ¿Tienes una reacción o pensamientos sobre lo que otros han compartido?

→ ¿Cuáles son algunas cosas que puedan ayudarse para superar estas barreras? (notetaker make a list!)
