# Supplementary material for: Stressors for farmworker parents during wildfire season
Source: BMC Public Health. 2024 Nov 28;24:3314. doi: 10.1186/s12889-024-20671-x (PMC11603887; doi:10.1186/s12889-024-20671-x)
Supplement: Supplementary file 4 — Supplementary Material 4 [file 12889_2024_20671_MOESM4_ESM.pdf]

<u>Resource Organizations in attendance at townhall events</u>

Chelan

- Washington Department of Labor & Industries
- Community Health Network of Washington
- Epic Wenatchee
- Wenatchee CAFÉ
- Wenatchee Valley YMCA
- Quincy Community Health Center

Okanogan

- Community Health Network of Washington
- Clean Air Methow
- Northwest Justice Project
- Epic Bridgeport
- Wenatchee CAFÉ
