# Supplementary material for: Stressors for farmworker parents during wildfire season
Source: BMC Public Health. 2024 Nov 28;24:3314. doi: 10.1186/s12889-024-20671-x (PMC11603887; doi:10.1186/s12889-024-20671-x)
Supplement: Supplementary file 5 — Supplementary Material 5 [file 12889_2024_20671_MOESM5_ESM.docx]

**Additional documents**

Appendix A.

- PDF
- Interview Guide
- Detailed interview guide used to collect data from farmworker parents.

Appendix B

- PDF
- Resource Organizations
- List of the resource organizations or CBOs that attended the townhall events to share resources with farmworker families.

Appendix C

- PDF
- Demographics Survey
- Questions asked of all interview and townhall participants.

Appendix D.

- PDF
- Townhall Discussion Questions
- Facilitator’s Guide used to lead round table conversations with farmworker families during the townhall event.
